# Inhibition of Transcription Induces Phosphorylation of YB-1 at Ser102 and Its Accumulation in the Nucleus

**DOI:** 10.3390/cells9010104

**Published:** 2019-12-31

**Authors:** Dmitry A. Kretov, Daria A. Mordovkina, Irina A. Eliseeva, Dmitry N. Lyabin, Dmitry N. Polyakov, Vandana Joshi, Bénédicte Desforges, Loic Hamon, Olga I. Lavrik, David Pastré, Patrick A. Curmi, Lev P. Ovchinnikov

**Affiliations:** 1 Institute of Protein Research, Russian Academy of Sciences, Pushchino 142290, Russia; dkretov@bu.edu (D.A.K.); darja-mordovkina@rambler.ru (D.A.M.); yeliseeva@vega.protres.ru (I.A.E.); lyabin@vega.protres.ru (D.N.L.); dmitry-polyakov@rambler.ru (D.N.P.); 2 SABNP, Univ.Evry, INSERM U1204, Université Paris-Saclay, 91025 Evry, France; vandana.joshi@univ-evry.fr (V.J.); benedicte.desforges@univ-evry.fr (B.D.); loic.hamon@univ-evry.fr (L.H.); david.pastre@univ-evry.fr (D.P.); 3 Institute of Chemical Biology and Fundamental Medicine, Siberian Branch of Russian Academy of Sciences (SB RAS), Novosibirsk 630090, Russia; lavrik@niboch.nsc.ru

**Keywords:** YB-1, nuclear translocation, inhibition of transcription, phosphorylation

## Abstract

The Y-box binding protein 1 (YB-1) is an RNA/DNA-binding protein regulating gene expression in the cytoplasm and the nucleus. Although mostly cytoplasmic, YB-1 accumulates in the nucleus under stress conditions. Its nuclear localization is associated with aggressiveness and multidrug resistance of cancer cells, which makes the understanding of the regulatory mechanisms of YB-1 subcellular distribution essential. Here, we report that inhibition of RNA polymerase II (RNAPII) activity results in the nuclear accumulation of YB-1 accompanied by its phosphorylation at Ser102. The inhibition of kinase activity reduces YB-1 phosphorylation and its accumulation in the nucleus. The presence of RNA in the nucleus is shown to be required for the nuclear retention of YB-1. Thus, the subcellular localization of YB-1 depends on its post-translational modifications (PTMs) and intracellular RNA distribution.

## 1. Introduction

The Y-box binding protein 1 (YB-1) is an RNA/DNA-binding protein that regulates gene expression in both the nucleus and the cytoplasm [1,2]. In the cytoplasm, YB-1 binds to mRNA, thereby regulating its stability and accessibility to the translational machinery [3,4,5]. In addition, YB-1 participates in the metabolism of non-coding RNAs in this cell compartment [6,7]. In the nucleus, YB-1 was initially believed to play the role of a transcription factor binding to Y-box elements in the promoter regions of some genes [8]. However, recent reports indicate that YB-1 does not display any preference towards Y-boxes both in vitro [9] and in vivo [10], and its role in the regulation of transcription could be better explained through its interaction with pre-mRNAs [11]. In agreement with this finding, it was demonstrated that YB-1 is recruited to nascent mRNAs during transcription [12]. Moreover, YB-1 was identified as a component of the spliceosome, which plays a role in alternative splicing affecting the inclusion or skipping of exons [13,14,15]. Additionally, there is evidence indicating the involvement of YB-1 in DNA repair [16,17,18,19,20].

Since YB-1 performs its functions in both the nucleus and the cytoplasm, its intracellular distribution must be tightly regulated to ensure that the proper amount of YB-1 is present in each cell compartment. Interestingly, in unstressed cells, YB-1 is detected predominantly in the cytoplasm, but it accumulates in the nucleus under certain conditions. For example, YB-1 is observed in the nucleus at the G_1_/S boundary of the cell cycle [21] and upon cell treatment with growth factors [22,23] or DNA-damaging drugs [24,25,26]. Moreover, a larger amount of nuclear YB-1 was observed to correlate with increased tumor malignancy and the acquisition of multiple drug resistance (MDR) in various cancers [27,28,29,30].

The intracellular distribution of YB-1 is regulated by two motifs present in its sequence, namely the nuclear localization signal (NLS) (aa 186–205) and the cytoplasmic retention signal (CRS) (aa 267–293) [21,31,32]. Under normal conditions, the effect of CRS dominates over NLS, thus ensuring cytoplasmic localization of YB-1. Nuclear translocation of YB-1 can be triggered either by its interaction with some protein partners or by its post-translational modifications (PTMs) [23,24,33,34]. For example, phosphorylation of YB-1 at Ser102, Ser165, Ser176, and Tyr281 and its cleavage by 20S proteasome can result in its nuclear translocation [24,31,35,36,37,38,39]. The transport of YB-1 through a nuclear pore is mediated by transportin 1 [40,41].

The current study shows that direct inhibition of the activity of RNA polymerase II (RNAPII) results in the gradual accumulation of YB-1 in the nucleus, which correlates with a decreasing level of cytoplasmic mRNAs. Furthermore, inhibition of transcription results in the activation of Akt kinase and phosphorylation of YB-1 at Ser102. Interestingly, with inhibited transcription, the kinase inhibitors caffeine and wortmannin inhibit phosphorylation of YB-1 and impair its nuclear accumulation. Lastly, we demonstrate that the nuclear retention of YB-1 depends on the RNA presence in this compartment. Together, these results indicate that the nuclear accumulation of YB-1 in response to the transcription inhibition is a sophisticated process that requires a decrease in the cytoplasmic mRNA level and phosphorylation of YB-1 at Ser102.

## 2. Materials and Methods

### 2.1. Cell Lines and Treatments

The Vero, NIH3T3, and MCF7 cell lines were obtained from the ATCC. They were cultivated in Dulbecco’s modified Eagle’s medium (DMEM) supplemented with 5% (*v*/*v*) fetal bovine serum (FBS), 2 mM L-glutamine, and 1% antibiotics (penicillin and streptomycin) in a humidified 5% CO_2_ atmosphere at 37 °C. Cells were treated with DMSO (control, 0.1%), actinomycin D (ActD) (5 μg/mL), 5,6-dichlorobenzimidazole 1-β-d-ribofuranoside(DRB) (100 μM), flavopiridol (1 μM) and α-amanitin (2 μg/mL), cycloheximide (10 μM), caffeine (10 mM), and wortmannin (0.5 μM) for indicated times or with sodium arsenite (300 μM) for 45 min to induce the formation of stress granules. All compounds were obtained from Sigma-Aldrich (Saint Louis, MO, USA).

### 2.2. Immunofluorescence Microscopy

Cells were fixed with 4% PFA in PBS for 20 min at 37 °C. After fixation, cells were washed and incubated for 12 h at 4 °C with rabbit polyclonal anti-YB-1 antibodies and anti-γH2AX, H3 antibodies in blocking solution (Tris-HCl pH 8.0, NaCl 100 mM, bovine serum albumin (BSA) 2%, Triton X-100 0.15%). The cells were then washed three times with PBS, after which appropriate secondary antibodies in blocking solution were added for 1 h at room temperature (RT). After final washes with PBS, the samples were mounted for fluorescence microscopy imaging. For DNaseI and RNaseA treatments, the cells were incubated with cytoskeleton (CSK) buffer (10 mM PIPES, pH 6.8, 100 mM NaCl, 3 mM MgCl_2_, 0.5% Triton X-100, 300 mM sucrose) before fixation containing 0.5 U/μL DNaseI or 5 μg/mL RNaseA, respectively, for 30 min at 37 °C.

### 2.3. In Situ Hybridization

After fixation with 4% PFA, the cells were successively incubated in 100% methanol and in 70% ethanol for at least 10 min. Then, they were placed in 1M Tris-HCl, pH 8.0, for 5 min. Cy3-conjugated oligo(dT)40 probe (1 μg/mL) (Sigma-Aldrich) was added to hybridization buffer (0.005% BSA, 1 mg/mL yeast RNA, 10% dextran sulfate, 25% formamide in 2XSSC (300 mM NaCl, 30 mM trisodium citrate)), and the coverslips were then placed in a humidity chamber for 1 h at 37 °C. Following the hybridization, the cells were rinsed once with 4XSSC and twice with 2XSSC. For additional immunofluorescence, after in situ hybridization, primary antibodies were diluted in 2XSSC supplemented with 0.1% Triton X-100 and incubated overnight at 4 °C. Then, the cells were washed three times, and the appropriate secondary antibody diluted in 2XSSC + 0.1% Triton X-100 was applied. The cells were incubated for 1 h at RT before three final washes in 2XSSC, mounted, and observed as described above. For rRNA hybridization, the following probes were used: 18S probe 5’-AAGGATTTAAAGTGGACTCATTCCAATTAC and 28S probe 5’-GGATTCTGACTTAGAGGCGTTCAGTCATAA, conjugated with Cy3.

### 2.4. Western Blotting

Denatured protein samples were separated by electrophoresis in 12% SDS-PAGE, transferred onto nitrocellulose membranes, blocked with 5% BSA in TBS-tween (TBST), washed with TBST, and incubated with primary antibodies diluted in 5% BSA in TBST overnight at 4 °C. Primary antibodies were detected using HRP-conjugated secondary antibodies diluted in 5% non-fat milk in TBST for 2 h at RT.

### 2.5. Antibodies

Anti-YB-1 (A303-231A, Bethyl (Montgomery, TX, USA), Rabbit Polyclonal) used at 1:10,000; anti-pYB-1 (Phospho-YB1 (Ser102) (C34A2) Rabbit mAb #2900, Cell Signaling (Danvers, MA, USA)) used at 1:2000; anti-pAkt (Phospho-Akt (Ser473) (D9E) Rabbit mAb #4060, Cell Signaling) used at 1:2000; anti-total Akt (Akt (pan) (C67E7) Rabbit mAb #4691, Cell Signaling) used at 1:2000; anti-H3 (Histone H3 (D1H2) Rabbit mAb #4499, Cell Signaling) used at 1:2000; anti-pS6 (Phospho-S6 Ribosomal Protein (Ser235/236) (D57.2.2E) Rabbit mAb #4858, Cell Signaling) used at 1:2000; anti-S6 (S6 Ribosomal Protein (5G10) Rabbit mAb #2217, Cell Signaling) used at 1:2000; anti-p GSK-3β(Phospho-GSK-3β (Ser9) (D85E12) Rabbit mAb #5558, Cell Signaling) used at 1:2000; anti-γH2AX (Anti-phospho-Histone H2A.X (Ser139) Antibody, clone JBW301, Millipore (Temecula, CA, USA); anti-rabbit: (Anti-rabbit IgG, HRP-linked antibody #7074, Cell Signaling,) used at 1:4000; anti-mouse (Anti-Mouse IgG, HRP-linked antibody, A9044, Sigma-Aldrich) used at 1:10,000 for Western blotting.

## 3. Results

### 3.1. Inhibition of RNAPII Induces the Nuclear Accumulation of YB-1

Several studies have shown that DNA-damaging drugs could provoke the nuclear translocation of YB-1 in several cell lines [24,26,42,43]. Additionally, a higher level of nuclear YB-1 was detected in samples from patients with advanced cancer who were undergoing chemotherapy [27,44,45]. These observations evidenced that damaged DNA could trigger YB-1 translocation to the nucleus. Importantly, the drugs used in these studies, apart from their DNA-damaging ability, may also affect the transcriptional activity of the cell [46]. Therefore, we aimed to reveal whether the nuclear accumulation of YB-1 results from the drug-induced DNA damage and/or it is a consequence of the induced transcriptional arrest.

**Figure 1:**
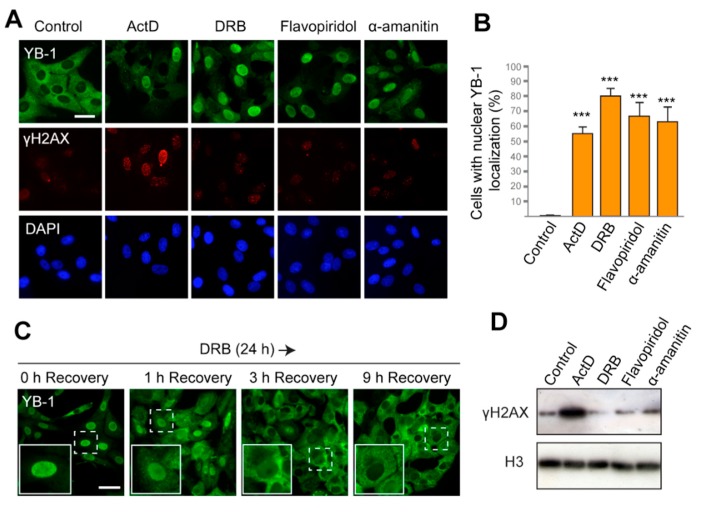
The Y-box binding protein 1 (YB-1) accumulates in the nucleus in response to inhibition of transcription. (**A**) Localization of YB-1 in Vero cells treated with DMSO (control), actinomycin D (ActD), 5,6-dichlorobenzimidazole 1-β-D-ribofuranoside(DRB), flavopiridol, and α-amanitin for 24 h. Scale bar 50 μm. (**B**) Quantification of cells with predominant nuclear localization of YB-1 (signal in the nucleus is higher than that in the cytoplasm) after 24 h treatment with ActD, DRB, flavopiridol, or α-amanitin. Results are mean ± SD. *** *p* < 0.001, paired *t*-test; *n* = 50 for each condition in three independent experiments. Examples of images used for quantification are shown in Figure S1A. (**C**) Localization of YB-1 in Vero cells after treatment with DRB for 24 h, followed by withdrawal of the drug (1, 3, and 9 h time points of recovery are indicated). Scale bar 50 μm. (**D**) Western blot for histone γH2AX and histone H3 of the whole-cell extract from Vero cells treated with ActD, DRB, flavopiridol, and α-amanitin for 24 h.

First, we used actinomycin D (ActD), a DNA-intercalating agent that generates double-strand breaks (DSBs) in DNA and blocks elongation of transcription [47,48]. The ability of ActD to induce nuclear translocation of YB-1 was previously reported [42,49,50] and was also reproduced in our experimental system (Figure 1A,B; Figures S1A and S7, Supplementary Materials). In addition to ActD, we tested three other compounds that can efficiently block transcription but induce no DNA damage. 5,6-Dichlorobenzimidazole 1-β-D-ribofuranoside(DRB) and flavopiridol are inhibitors of the CDK9 kinase, whose inhibition prevents phosphorylation of the C-terminal domain of RNAPII, thereby providing cessation of transcription. Another drug, α-amanitin, binds with high affinity to the active site of RNAPII and impairs its translocation along DNA after nucleotide addition [51,52]. We observed that the majority of cells treated with DRB, flavopiridol, and α-amanitin display the nuclear localization of YB-1 (Figure 1A,B and Figure S1A). Since α-amanitin induces nuclear translocation of YB-1, we can conclude that inhibition of RNAPII, but not RNAPI and RNAPIII, is required for this translocation. Cycloheximide does not prevent the nuclear accumulation of YB-1, thus indicating that de novo translation is not required, and it is likely that the presynthesized YB-1 is transferred to the nucleus (Figure S1B, Supplementary Materials). Interestingly, translocation of YB-1 from the cytoplasm to the nucleus was also found to be a gradual process that takes several hours after transcription blockage (Figure S2A, Supplementary Materials).

To assess the level of DNA damage in the treated cells, we visualized the phosphorylated histone H2AX (γH2AX), which specifically marks DSBs in the nucleus. As expected, ActD induced strong phosphorylation of H2AX (Figure 1A,D). However, with other transcription inhibitors used, no γH2AX foci were detected. This observation indicates that the nuclear localization of YB-1 induced by DRB, flavopiridol, and α-amanitin occurs in the absence of DSBs.

If transcription inhibition is the primary reason for the nuclear accumulation of YB-1, the restoration of its cytoplasmic localization can be expected to occur upon the cessation of transcription blockage. ActD and α-amanitin are irreversible inhibitors of transcription and, therefore, they could not be used to verify this hypothesis. However, the action of DRB and flavopiridol could be reverted, thus allowing the resumption of transcription [48]. Therefore, we exposed cells to DRB for 24 h and then withdrew the drug. The intracellular localization of YB-1 was monitored at different time intervals during the recovery (Figure 1C and Figure S2B,C). Strikingly, as early as 1 h after DRB removal, YB-1 started accumulating in the cytoplasm. After 6 h, the cytoplasmic accumulation appeared more pronounced, and after 9 h, the majority of the cells had YB-1 mostly in the cytoplasm. Similar results were observed when cells were treated with flavopiridol and then transferred to a drug-free medium (Figure S2C, Supplementary Materials). These observations further underscore the hypothesis that the intracellular localization/distribution of YB-1 is a dynamic process that strongly depends on the activity of the transcriptional machinery.

### 3.2. Inhibition of RNAPII Affects Distribution of Poly(A^+^)RNA in the Cell

**Figure 2:**
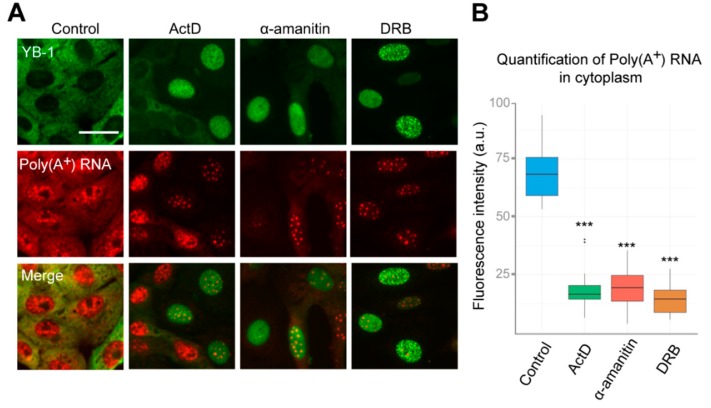
Inhibition of RNAPII affects distribution of poly(A^+^)RNA in the cell. (**A**) Intracellular localization of YB-1 and poly(A^+^) after in situ hybridization in Vero cells exposed to DMSO (control), DRB, ActD, and α-amanitin for 24 h. Scale bar 50 μm. (**B**) Quantification of cytoplasmic poly(A^+^)RNA levels in control cells or exposed to the inhibitors of transcription. Results are mean ± SD. *** *p* < 0.001, paired *t*-test; *n* = 25 for each condition in three independent experiments.

YB-1 is one of the most abundant mRNA-binding proteins in the cytoplasm; it binds to mRNA with high affinity and participates in the regulation of its translation and stability [1,2]. Changes in mRNA distribution can strongly affect the YB-1 localization, as demonstrated by YB-1 re-localizing into stress granules when the cells are exposed to oxidative stress (Figure S3A, Supplementary Materials) [53]. In this aspect, inhibition of transcription may have a dramatic impact on mRNA distribution within the cell, thereby potentially contributing to changes in the localization of YB-1. Therefore, we evaluated the dynamics of mRNA distribution in the cell in our experimental conditions. We performed in situ hybridization using fluorescently labeled oligo(dT) probes recognizing poly(A) tails of mRNAs. Interestingly, when the cells were treated with ActD, DRB, or α-amanitin for 24 h, we observed a strong decrease in poly(A^+^)mRNA levels in the cytoplasm (Figure 2A,B). Indeed, the median half-life of mRNA is about 12 h, as measured in mammalian cells [54], which indicates that more than 50%–70% of mRNAs should be degraded after 24 h transcription blockage. At the same time, no visible decrease of ribosomal RNA levels was detected under these conditions, which can be explained by greater stability of RNA, as compared to mRNA (Figure S3B, Supplementary Materials). Therefore, this experiment revealed that the degradation of mRNA in the cytoplasm correlates with YB-1 translocation to the nucleus.

**Figure 3:**
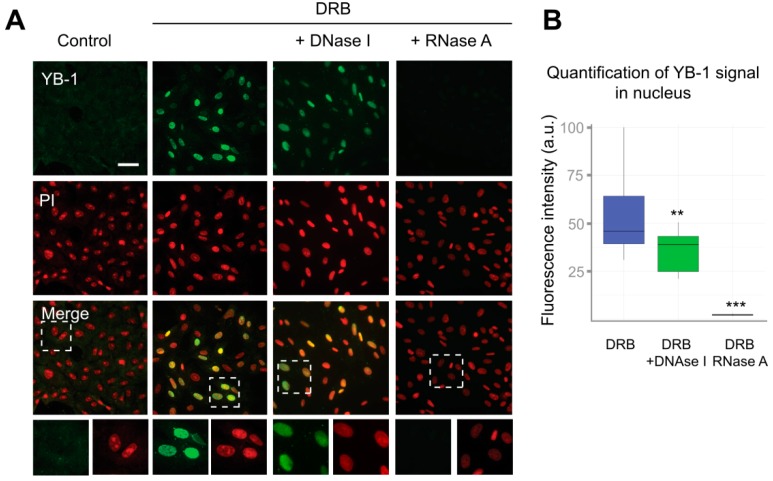
RNA is required for the retention of YB-1 in the nucleus. (**A**) Control (DMSO-treated) Vero cells or cells exposed to DRB for 24 h were washed with CSK buffer prior to fixation. In the case of DRB-treated cells, either DNA or RNA was additionally digested with DNaseI or RNaseA, respectively. Scale bar 50 μm. (**B**) Quantification of retention of the YB-1 signal in the nuclei of cells treated with DRB that were freshly permeabilized or treated with DNaseI or RNaseA. Results are mean ± SD. ** *p* < 0.01, *** *p* < 0.001, paired *t*-test; *n* = 15 for each condition.

Interestingly, the distribution of poly(A^+^) RNA within the nucleus was also significantly affected in cells treated with inhibitors of transcription, as compared to control cells. Poly(A^+^) RNA was detected in the large nuclear speckles that serve to store pre-mRNA [48]. However, YB-1 does not colocalize with these speckles having rather diffuse distribution in the nucleus (Figure 2A). This indicates that, in the nucleus, YB-1 most likely interacts not only with poly(A^+^) RNA but also with other types of RNA, DNA, or protein partners. To clarify whether some strong interactions mediate nuclear retention of YB-1, we permeabilized the cells with buffer containing detergent (CSK) prior to fixation causing the removal of all proteins that are not strongly anchored in the nucleus. In control cells (without transcription inhibitors), no YB-1 signal was detected (Figure S4A, Supplementary Materials). Indeed, under these conditions, YB-1 was absent from the nucleus showing utterly cytoplasmic localization (Figure 1A and Figure 2A) that would not withstand the detergent treatment. However, in the cells treated with ActD, DRB, flavopiridol, and α-amanitin, YB-1 remained in the nucleus in the presence of the CSK buffer (Figure 3A and Figure S4A,B), thus showing that it is anchored there by tight binding to some of its partners.

Although YB-1 exhibits a higher affinity for RNA than for DNA, it is capable of binding to both of them [55,56]. To identify the type of nucleic acids to which YB-1 binds in the nucleus, we performed separate digestion of DNA and RNA using the CSK buffer containing either DNaseI or RNaseA. To visualize DNA or RNA after their separate digestion, we used propidium iodide (PI) that efficiently binds to both RNA and DNA. After DNA digestion, we observed the characteristic PI staining indicative of abundant ribosomal RNA (rRNA), which showed that DNA had been successfully removed from the nucleus (Figure 3A and Figure S4B). In contrast, in RNaseA-treated cells, the PI staining was rather homogeneous without detectable nucleoli (Figure S4B, Supplementary Materials). Interestingly, the reduction of the nuclear presence of YB-1 in DNaseI-treated cells was relatively modest. In contrast, the removal of RNA resulted in the complete disappearance of the YB-1 signal (Figure 3A,B). These experiments suggest that RNA, but not DNA, is crucially required to anchor YB-1 in the nucleus.

### 3.3. Inhibition of Transcription Entails YB-1 Phosphorylation at Ser102

**Figure 4:**
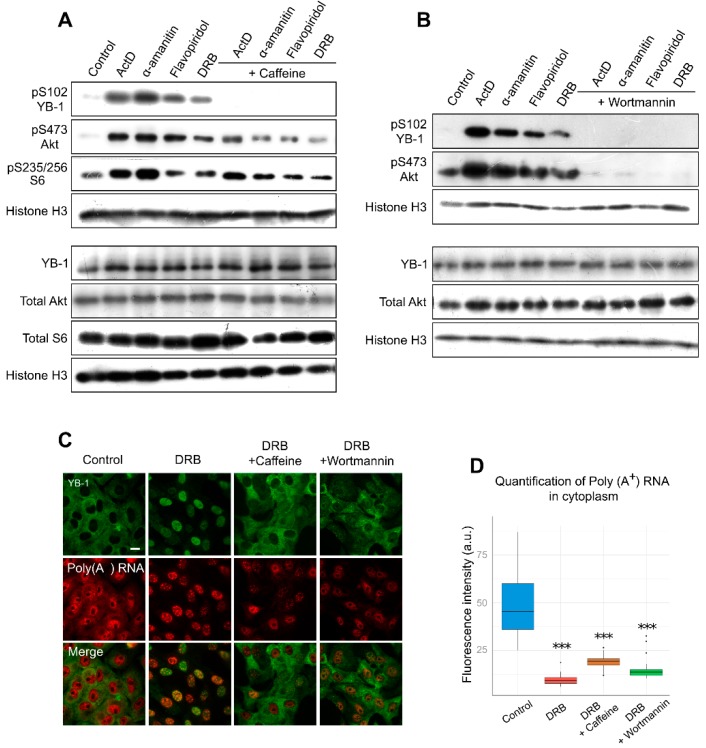
Inhibition of transcription entails YB-1 phosphorylation at Ser102. (**A**,**B**). Western blot of the whole extracts from Vero cells treated with ActD, flavopiridol, α-amanitin, and DRB for 24 h in the presence or absence of caffeine (**A**) or wortmannin (**B**). (**C**) Immunofluorescence microscopy of YB-1 and in situ hybridization of poly(A^+^) RNA in Vero cells treated with DRB in the presence of caffeine or wortmannin. Scale bar 50 μm. (**D**) Quantification of cytoplasmic poly(A^+^) RNA in control cells or in cells treated with DRB alone or DRB together with caffeine or wortmannin. Results are mean ± SD. *** *p* < 0.001, paired *t*-test; *n* = 20 for each condition.

Several post-translational modifications are associated with the nuclear accumulation of YB-1. Phosphorylation is one of the best-characterized modifications of YB-1, which occurs at several positions and has been reported to affect its intracellular localization [31,35,36,37]. Several studies have particularly highlighted the importance of YB-1 phosphorylation at Ser102 for its accumulation in the nucleus [22,35,38]. Therefore, we decided to investigate whether YB-1 is modified at Ser102 when cells are exposed to transcription inhibitors. Interestingly, all transcription inhibitors, including ActD, α-amanitin, flavopiridol, and DRB, appeared to induce Ser102 phosphorylation of YB-1, as compared to untreated cells (Figure 4A).

To estimate the potential impact of phosphorylation on the nuclear translocation of YB-1, we analyzed the effect of kinase inhibition on YB-1 localization in Vero cells using caffeine, a wide-range inhibitor [57,58]. We observed a lower level of YB-1 phosphorylation in the presence of both caffeine and transcription inhibitors (Figure 4A). Furthermore, caffeine presence strongly impaired the nuclear import of YB-1, and in most cells, YB-1 was retained in the cytoplasm (Figure 4C; Figure S5, Supplementary Materials). Of note, the same phenomenon was observed in NIH 3T3 cells (Figure S6, Supplementary Materials). We also assessed the mRNA level in these conditions and found that, in the cytoplasm, the mRNA level was strongly decreased despite the retention of YB-1 in this compartment (Figure 4D). This experiment demonstrates that, for the YB-1 translocation to the nucleus, the degradation of mRNAs in the cytoplasm alone is not sufficient; post-translational modifications of YB-1, such as its phosphorylation at Ser102, are additionally required.

It is known that Akt is one of the kinases that are responsible for YB-1 phosphorylation at Ser102 [35,38]. With the inhibited transcription, we observed an activation of Akt kinase, which correlated with YB-1 phosphorylation (Figure 4A,B). Interestingly, the simultaneous presence of DRB and wortmannin (PI3K/Akt pathway inhibitor) reduces YB-1 phosphorylation and nuclear accumulation without affecting the mRNA level in the cytoplasm. These results are in good agreement with the caffeine effect, so we can speculate that Akt might be responsible for YB-1 phosphorylation and nuclear accumulation upon transcription inhibition. Of note, wortmannin can inhibit not only Akt but also some other kinases [58]; therefore, we cannot rule out their involvement in this process.

## 4. Discussion

YB-1 regulates gene expression at multiple levels in the cell, exerting its functions both in the nucleus and in the cytoplasm. Under normal conditions in unstressed cells, YB-1 has a predominantly cytoplasmic localization where it plays a role in the regulation of mRNA translation and stability, as well as in the regulation of non-coding RNA metabolism [1,2,7]. Nonetheless, unbiased proteome-wide approaches have also detected YB-1 in the nucleus, thus indicating that a certain proportion of the protein is always present in this compartment [59]. Importantly, the shift of the balance from predominantly cytoplasmic to nuclear localization of YB-1 has been shown to lead to malignant transformation of cells, metastasis, and multidrug resistance [27,28,29]. Thus, YB-1 is a nucleo-cytoplasmic shuttling protein whose intracellular localization must be tightly regulated. Several mechanisms of nuclear translocation of YB-1 have been described, including its proteolytic cleavage by 20S proteasome [24] and phosphorylation at Ser102 [35] and Tyr281 [31]. Nonetheless, a detailed understanding of how the intracellular distribution of YB-1 is regulated within the cell and which factors may alter its localization is still lacking.

In the present study, we demonstrated that direct inhibition of RNAPII activity results in the nuclear accumulation of YB-1. Furthermore, we showed that the exposure of cells to transcription inhibitors results in a significant decrease of mRNA levels in the cytoplasm, which may lead to increased nuclear import of RNA-free YB-1. Moreover, we demonstrated that retention of YB-1 in the nucleus depends mostly on the presence of RNA in this compartment. Altogether, these findings indicate that RNA indeed plays a crucial role in the intracellular localization of YB-1. Additionally, the exposure of cells to transcription inhibitors results in the activation of Akt kinase and phosphorylation of YB-1 at Ser102. Kinase inhibitors, such as caffeine and wortmannin, are able to block phosphorylation of YB-1 at Ser102 and impair its nuclear accumulation. However, they do not prevent the degradation of cytoplasmic mRNAs when applied together with transcription inhibitors. This suggests that the mRNA degradation alone is not sufficient for the YB-1 import into the nucleus. A recent analysis of the global redistribution of RNA-binding proteins in the cell resulting from the viral ribonuclease-induced global RNA degradation revealed no increase in the YB-1 accumulation in the nucleus [60]. Phosphorylation of YB-1 may affect its interaction with mRNAs or play a role in its nuclear transport. For example, phosphorylation of YB-1 at Ser102 has been shown to decrease its affinity for the mRNA cap-structure [38]. On the other hand, it has been demonstrated that indirubin 3-oxime inhibits the nuclear accumulation of YB-1 in ActD- or DRB-treated cells [61]. The authors of that study proposed that the direct binding of indirubin 3-oxime to YB-1 leads to disruption of the YB-1 interaction with transportin 1 that mediates its transport through a nucleus pore [61]. However, indirubin 3-oxime is also known to inhibit the activity of several kinases, including Akt [62]. Hence, indirubin 3-oxime possibly interferes with YB-1 phosphorylation, thereby affecting the YB-1 translocation to the nucleus. Furthermore, it cannot be ruled out that phosphorylation at sites other than Ser102, or even other PTMs, occur under these conditions and may affect the YB-1 interaction with mRNAs, as well as with other factors that are important for its transport to the nucleus. Indeed, DRB induces the least pronounced YB-1 phosphorylation at Ser102, as well as the Akt activation, although it causes the most efficient YB-1 accumulation in the nucleus, thus indicating probable involvement of some additional mechanisms in the nuclear translocation of YB-1.

In the present study, we provide novel insights into the mechanism of YB-1 translocation to the nucleus in response to a decrease in RNAPII activity caused by specific inhibitors. The inhibition of RNAPII activity entails a lower level of cytoplasmic mRNA and the phosphorylation of YB-1 at Ser102. This may cause the appearance of abundant RNA-free YB-1 (previously associated with mRNAs as a major core mRNP protein) that interacts with transportin 1 and goes to the nucleus.

In the nucleus, YB-1 can stimulate transcription of many genes either as a transcription factor or as a co-factor. The YB-1 nuclear translocation caused by the presence of DNA-damaging xenobiotics may be realized by a similar mechanism because a DNA damage has been shown to lead to the global transcription blockage [46]. In this case, nuclear YB-1 can be involved in DNA repair [16,63,64,65]. Under physiological conditions with gradual degradation of cytoplasmic mRNAs, a portion of YB-1 may have the nuclear localization and maintain the optimal mRNA levels, both specific and global.

## Supplementary Materials

The following are available online at https://www.mdpi.com/2073-4409/9/1/104/s1, Figure S1: YB-1 localization after treatment with transcription inhibitors and cycloheximide. Figure S2: Dynamics of YB-1 localization after drug addition and removal. Figure S3: Localization of YB-1, poly(A^+^) RNA after treatment with NaAsO_2_; control to Figure 1 and Figure 2. Figure S4: Controls to Figure 3. Figure S5: YB-1 localization after treatment with transcription inhibitors and caffeine in Vero cells. Figure S6: YB-1 localization after treatment with transcription inhibitors and caffeine in NIH 3T3 cells. Figure S7: YB-1 localization after treatment with ActD in MCF7 cells.
