# Supplementary material for: Inhibition of Transcription Induces Phosphorylation of YB-1 at Ser102 and Its Accumulation in the Nucleus
Source: Cells. 2019 Dec 31;9(1):104. doi: 10.3390/cells9010104 (PMC7016903; doi:10.3390/cells9010104)
Supplement: Supplementary file 1 [file cells-09-00104-s001.pdf]

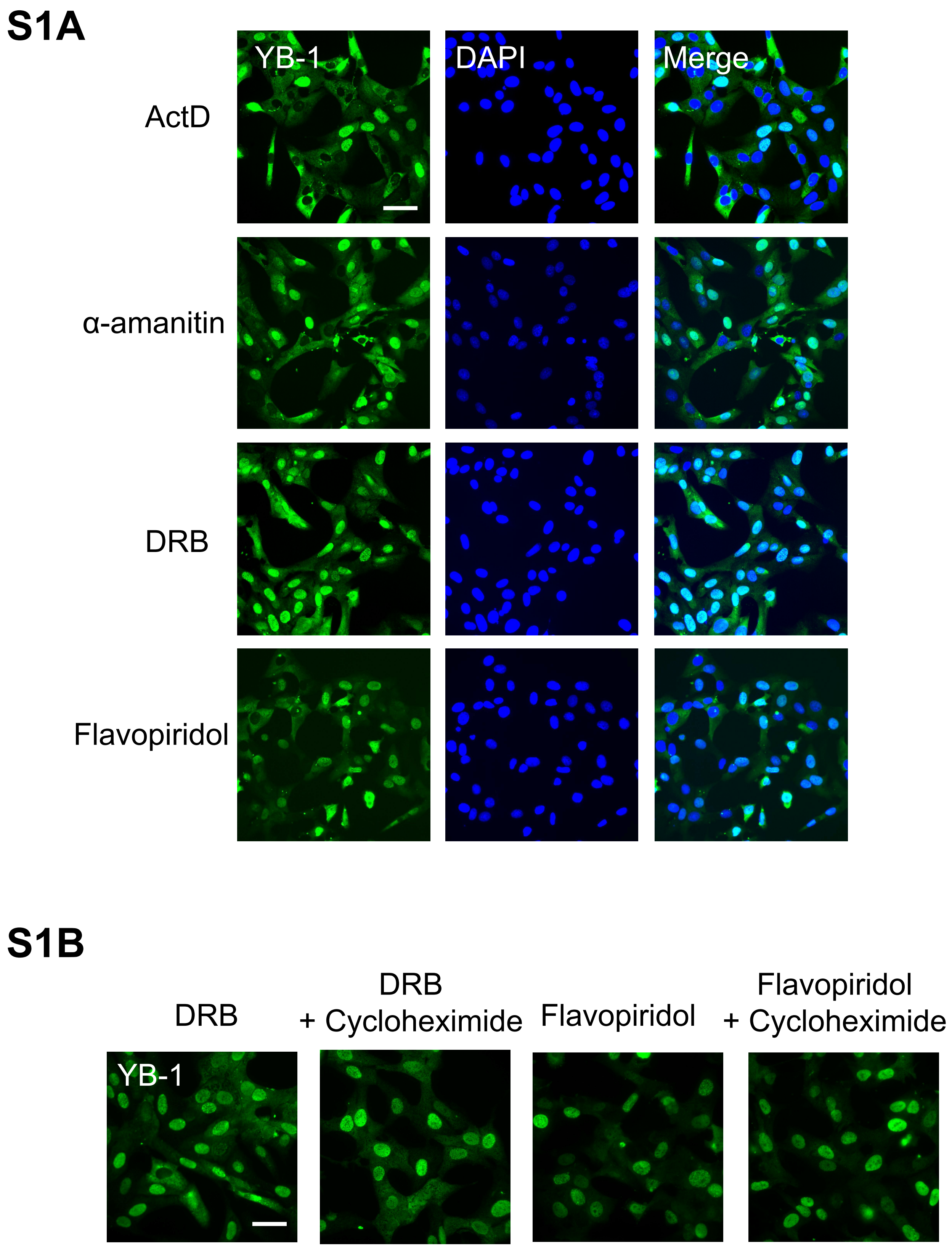


**FIGURE S1.**

**S1A)** Localization of YB-1 in Vero cells after treatment with ActD, DRB, flavopiridol, and α-amanitin for 24 h. These photos were used for quantification in Fig 1B; **S1B)** Vero cells were treated with DRB, flavopiridol alone or in the presence of cycloheximide.

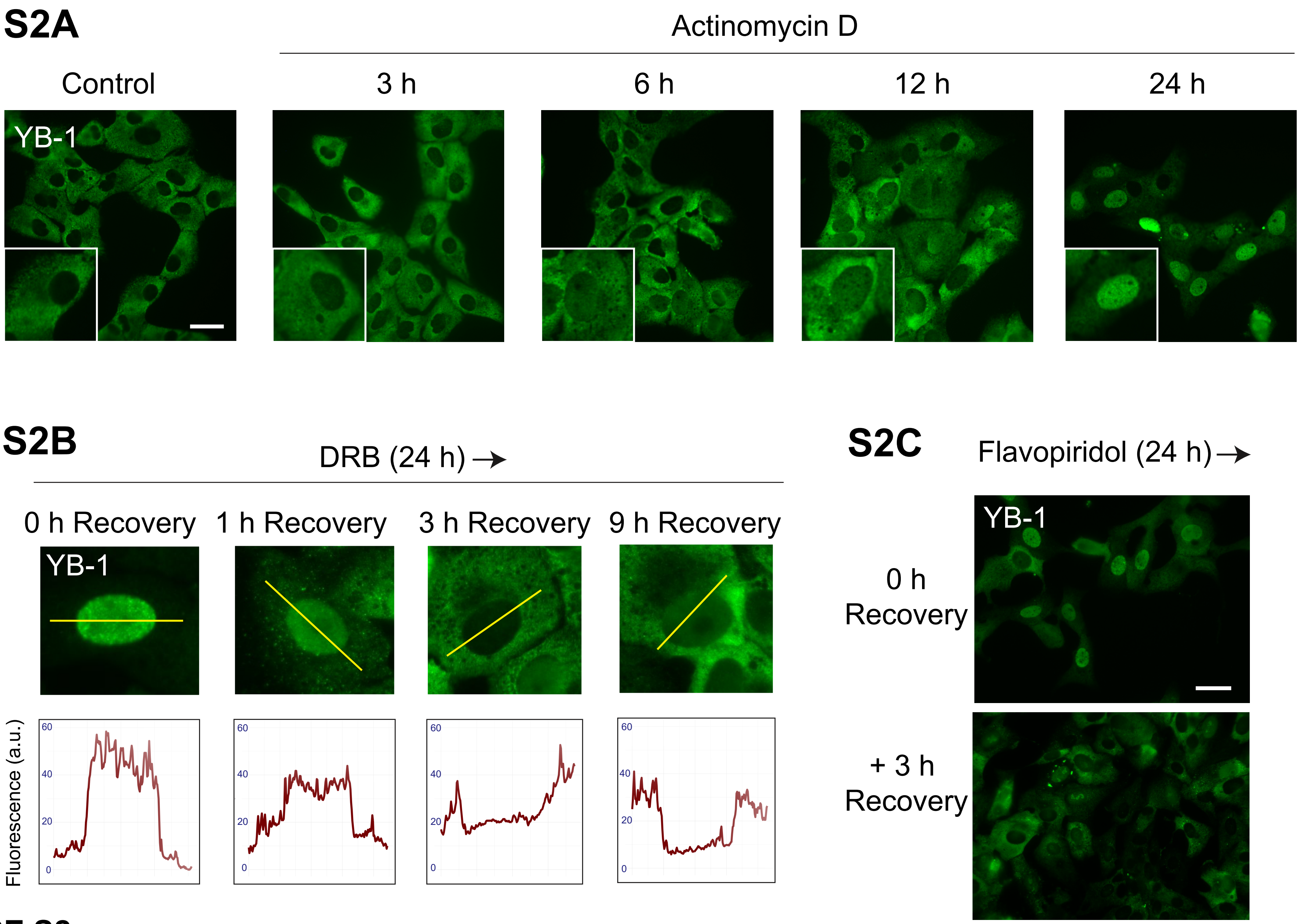


**FIGURE S2.**

**S2A)** Localization of YB-1 in Vero cells after treatment with ActD for 3, 6, 12 and 24 h; **S2B)** Re-localization of YB-1 from the nucleus to the cytoplasm in Vero cells recovering from DRB for 1, 3 and 9 h; **S2C)** Localization of YB-1 in Vero cells after treatment with flavopiridol for 24 h followed by 3 h drug withdrawn. Scale bar 50 μm.

## S3A

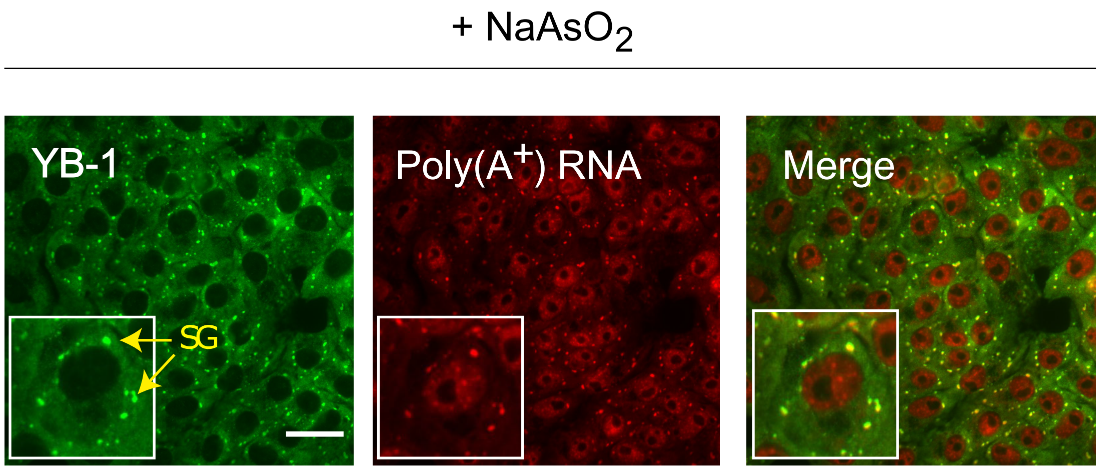


## S3B

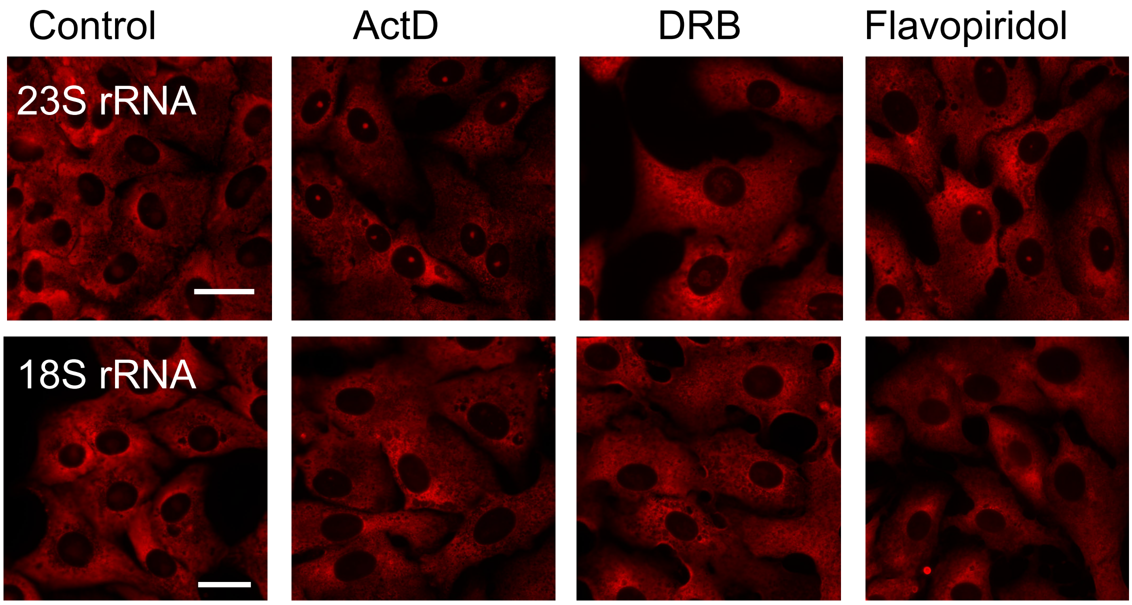


**FIGURE S3.**

**S3A)** Localization of YB-1 and poly($A^+$) RNA *in situ* hybridization in Vero cells treated with $NaAsO_2$ for 45 min; **S3B)** 28S and 18S ribosomal RNA *in situ* hybridization.

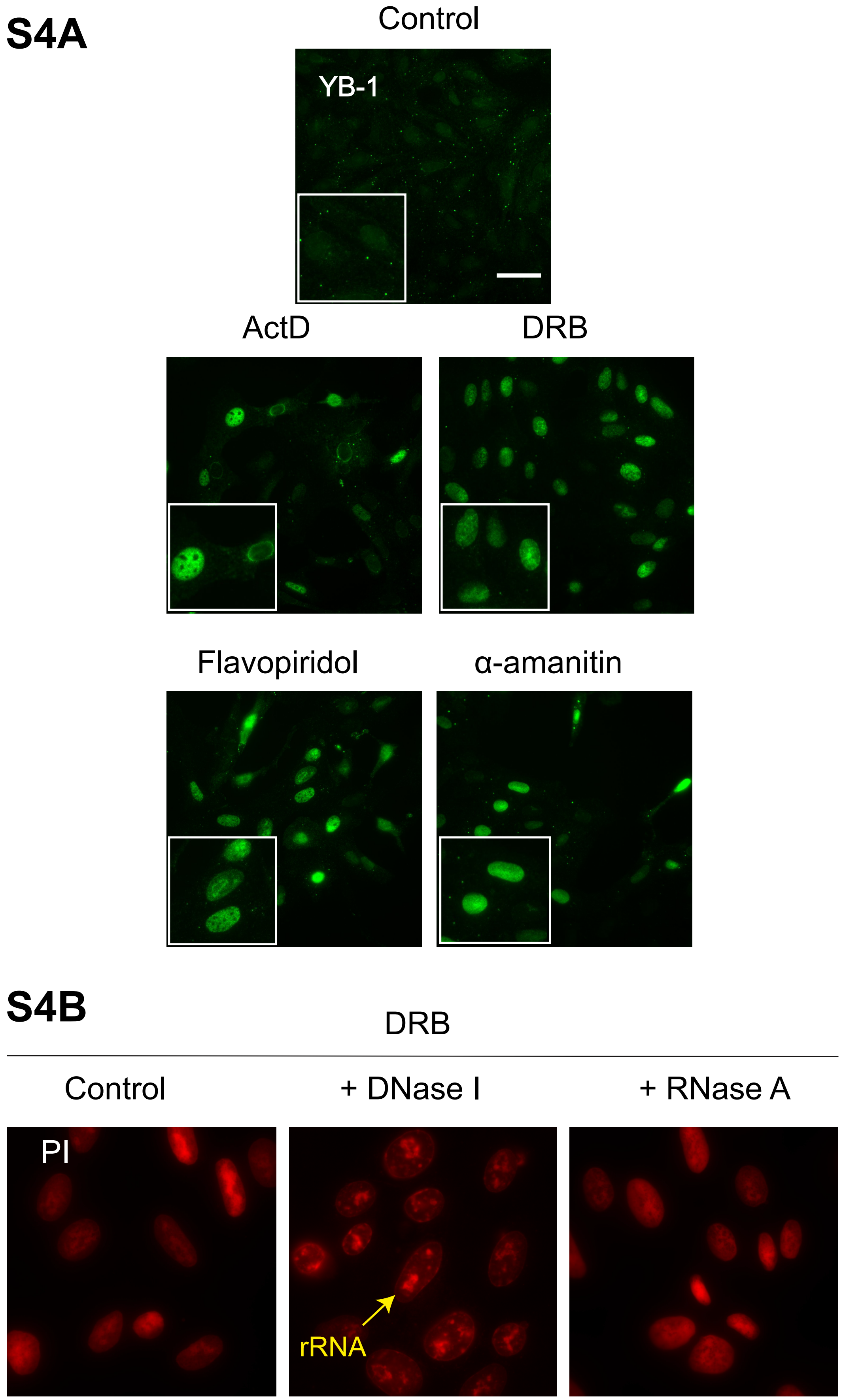


**FIGURE S4.**

**S4A)** Nuclear retention of YB-1 in Vero cells exposed to ActD, DRB, flavopiridol, and α-amanitin for 24 h and permeabilized with CSK buffer prior to fixation; **S4B)** Retention of YB-1 in the nuclei of cells treated with DRB and permeabilized with CSK buffer in the presence of DNaseI or RNaseA. Scale bar 50 μm.

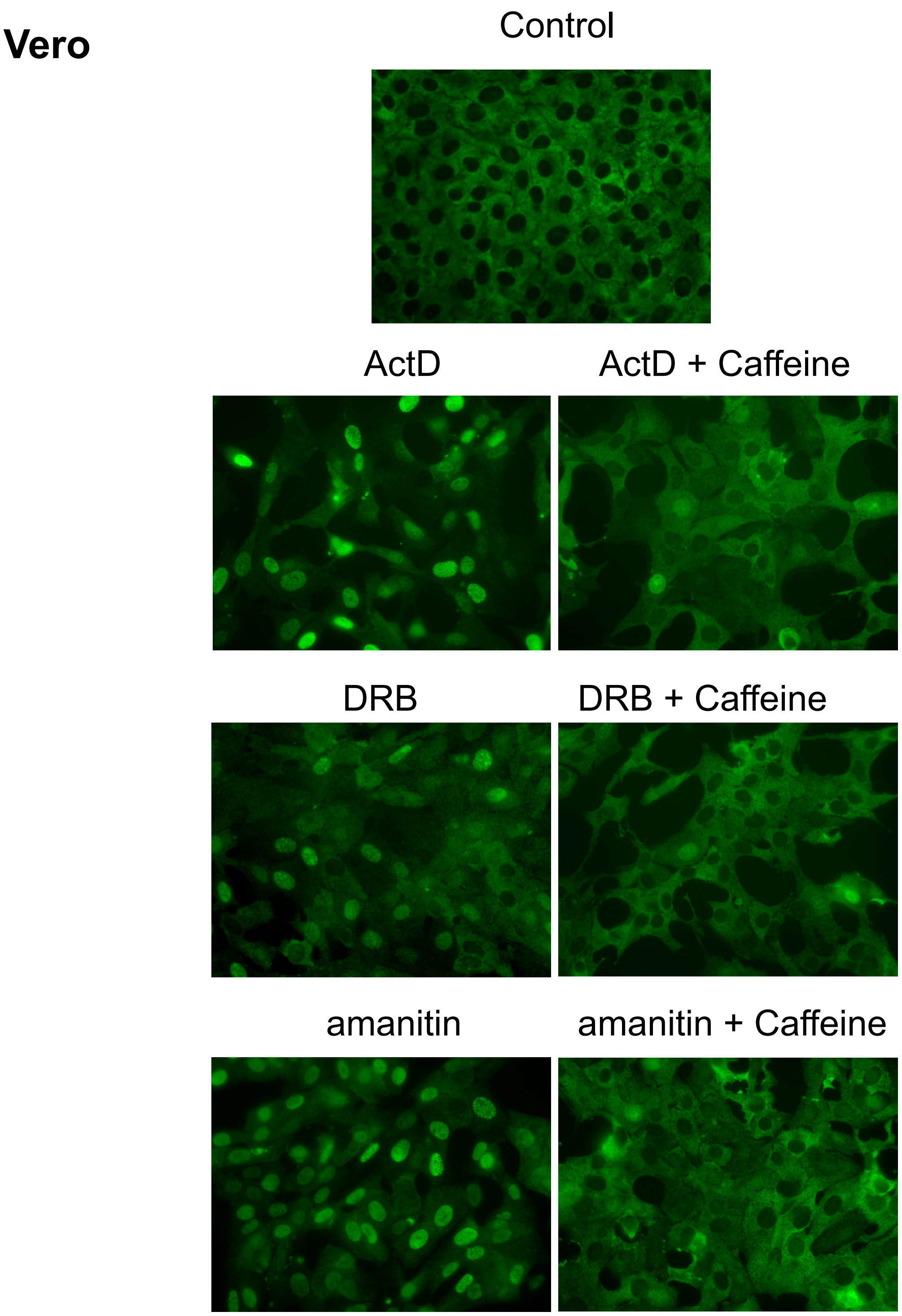


**FIGURE S5.**
Immunofluorescence microscopy of YB-1 in Vero cells treated with inhibitors of transcription in the presence or absence of caffeine.

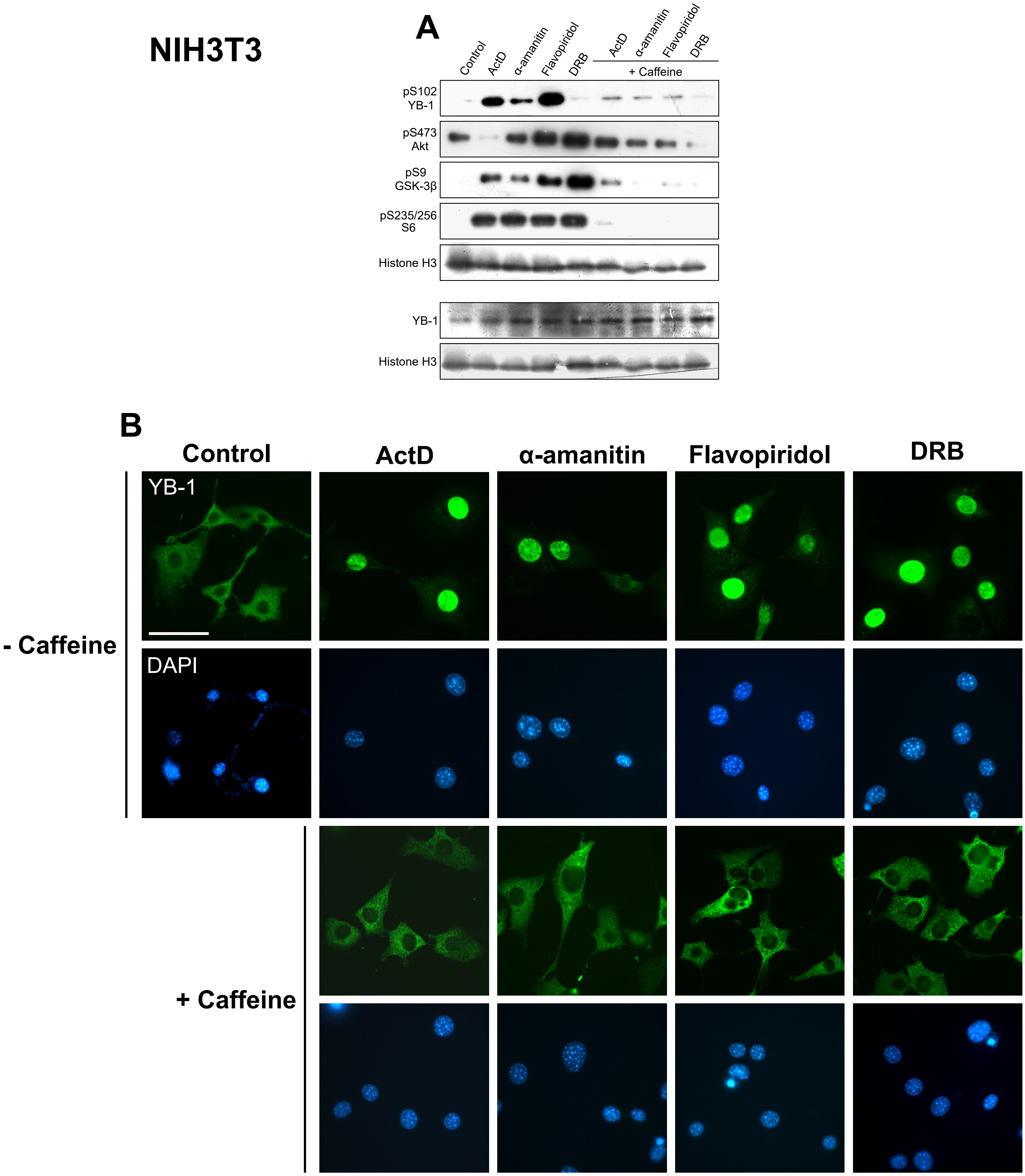


**FIGURE S6.**

**S6A)** Western blot of the whole extracts from NIH3T3 cells treated with ActD, flavopiridol, α-amanitin, and DRB for 24 h in the absence or the presence of caffeine; **S6B)** Immunofluorescence microscopy of YB-1 in NIH3T3 cells treated with inhibitors of transcription in the presence or absence of caffeine. Scale bar 50 μm.

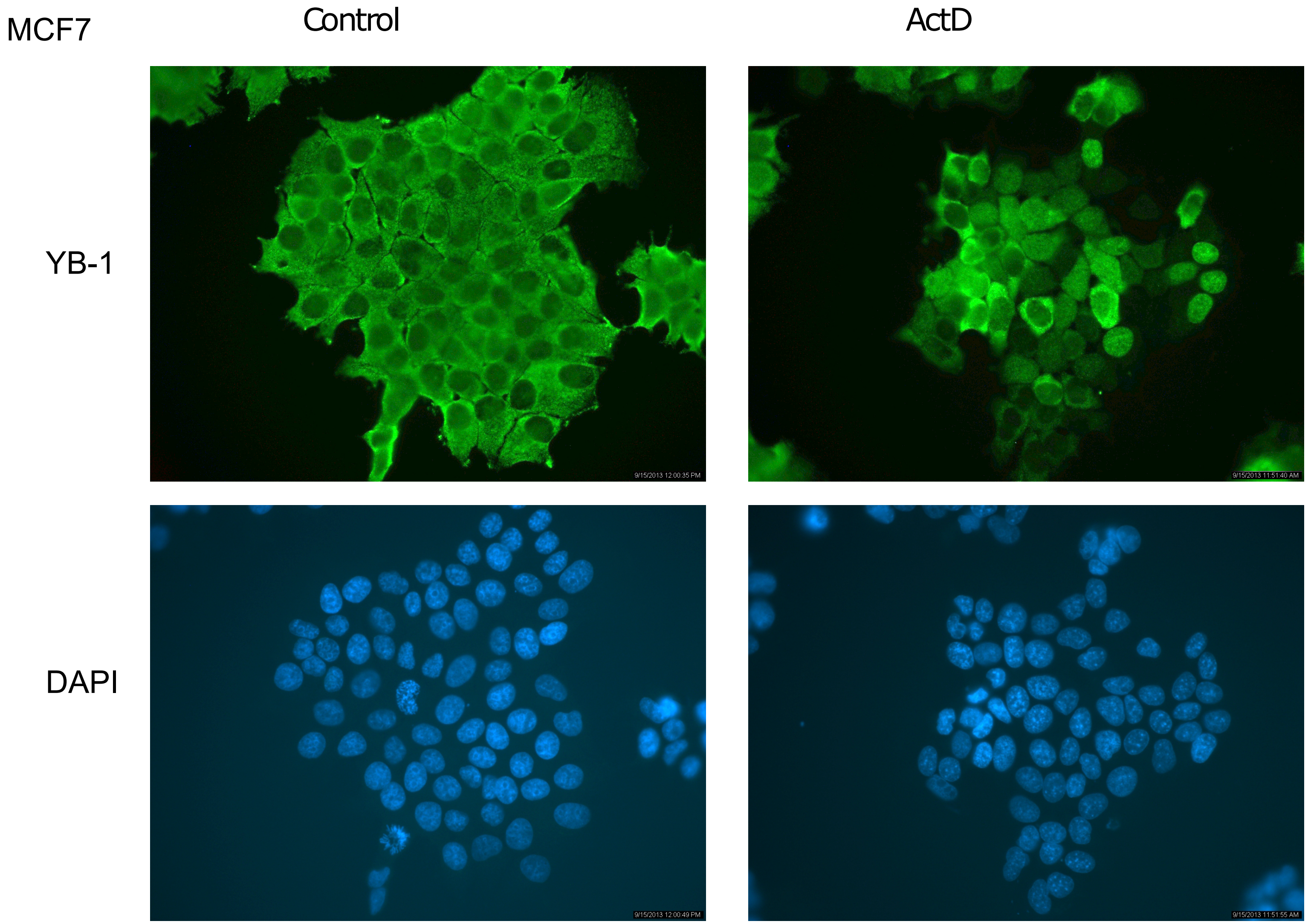


**FIGURE S7.**
Immunofluorescence microscopy of YB-1 in MCF7 cells treated with ActD.
